# Transcriptome Analysis of Zebrafish Olfactory Epithelium Reveal Sexual Differences in Odorant Detection

**DOI:** 10.3390/genes11060592

**Published:** 2020-05-27

**Authors:** Ying Wang, Haifeng Jiang, Liandong Yang

**Affiliations:** 1 Hubei Engineering Research Center for Protection and Utilization of Special Biological Resources in the Hanjiang River Basin, School of Life Sciences, Jianghan University, Wuhan 430056, Hubei, China; wangying1987@jhun.edu.cn; 2 The Key Laboratory of Aquatic Biodiversity and Conservation of Chinese Academy of Sciences, Institute of Hydrobiology, Chinese Academy of Sciences, Wuhan 430072, Hubei, China; jianghf@ihb.ac.cn; 3 University of Chinese Academy of Sciences, Beijing 100049, China

**Keywords:** zebrafish, sexual dimorphism, olfactory epithelium, alternative splicing, chemosensory receptor

## Abstract

Animals have evolved a large number of olfactory receptor genes in their genome to detect numerous odorants in their surrounding environments. However, we still know little about whether males and females possess the same abilities to sense odorants, especially in fish. In this study, we used deep RNA sequencing to examine the difference of transcriptome between male and female zebrafish olfactory epithelia. We found that the olfactory transcriptomes between males and females are highly similar. We also found evidence of some genes showing differential expression or alternative splicing, which may be associated with odorant-sensing between sexes. Most chemosensory receptor genes showed evidence of expression in the zebrafish olfactory epithelium, with a higher expression level in males than in females. Taken together, our results provide a comprehensive catalog of the genes mediating olfactory perception and pheromone-evoked behavior in fishes.

## 1. Introduction

Olfaction is a sense for detecting environmental odorants that plays essential roles in many aspects of animal activities, such as foraging, migration, prey avoidance and mating [1]. In most terrestrial vertebrates, there are two distinct chemosensory organs: the olfactory epithelium (OE) and the vomeronasal organ (VNO) [2]. In general, the OE is assumed to detect environmental odorants, while the VNO senses pheromones, although some exceptions were also reported in recent studies [3,4]. Different set of chemosensory receptors is thought to be expressed in each organ. For the OE, odorant receptors (ORs) and trace amine-associated receptors (TAARs) are known to be expressed, while for the VNO, vomeronasal receptors type 1 (V1Rs) and type 2 (V2Rs) are considered to be expressed [5]. Therefore, in terrestrial mammals, ORs and TAARs are suggested to detect "ordinary" odorant molecules, while V1Rs and V2Rs are employed to detect pheromones [6,7,8].

However, unlike the terrestrial counterparts, fish have only the OE to sense their olfactory environment, due to an absence of the VNO [9]. Without directly functional studies of the chemosensory receptors in fish, it is unclear whether the different types of chemosensory receptors in fish respond to different classes of odorants—and which family of the chemosensory receptors is used for recognizing odorants or pheromones. Interestingly, there are also some studies using electrophysiological experiments that provide indirect evidence of putative ligands for chemosensory receptors [10,11,12,13,14,15,16]. For example, members of ORs from goldfish can recognize F-prostaglandins, while both ORs and V2Rs can perceive amino acids [17,18].

Several studies have found that sex-specific behaviors can be due to sex differences in responses to external stimuli, including courtship songs, colors and chemosensory cues [19]. Indeed, chemosensory cues in mice, such as pheromone molecules, have been shown to be detected specifically by their vomeronasal receptors [20]. Therefore, sex-specific behaviors maybe initiated by sex-specific pheromones [21,22,23] or by sex-differential expression of chemosensory receptors in response to the same pheromones [24]—or even by sex-specific alternative splicing [25]. For instance, Darcin (the MUP20 encoded by Mup20)—a male-specific pheromone in mouse urine—only attracts females to affect their memory [24]. ESP1 (exocrine-secreted peptide 1) is detected by both male and female mice, but only stimulated female-specific mating behaviors, suggesting the existence of sex-specific neuronal circuits [23,26]. Male fruit flies require the protein encoded by the fruitless (fru) gene to complete courtship, which is produced in different sex-specific isoforms via alternative splicing [25]. However, to date, few studies have reported whether sexual differences in odorant-sensing exists between males and females, especially in fish.

To determine the full repertoire of chemosensory receptors expressed in the zebrafish olfactory epithelium and evaluate the extent of sexual differentiation in odorant-sensing between sexes, we used RNAseq to profile their transcriptomes in male and female zebrafish. We found that a very high percentage of chemosensory receptors are indeed expressed in the zebrafish olfactory epithelium, with a higher expression level in males than females. However, the olfactory transcriptomes between males and females are highly similar, with limited genes showing differentially expressed or alternatively splicing, which may be associated with odorant-sensing between sexes. Collectively, our results provide a comprehensive catalog of the genes mediating olfactory perception and pheromone-evoked behavior in fish.

## 2. Materials and Methods

### 2.1. Sample Collection

All experiments in this research were approved by the Institutional Animal Care and Use Committee of Institute of Hydrobiology, Chinese Academy of Sciences (Approval ID: Y21304501). Adult wild-type zebrafish from AB background were maintained in the zebrafish facilities in the China Zebrafish Resource Center (CZRC)for one week to familiarize with the laboratory environment. Zebrafish were raised together in a mixed sex population. Olfactory epithelium from each individual was dissected out and frozen in liquid nitrogen quickly. Due to their small size, each of the samples was a pool of mRNA from three individuals of the same sex. Three independent biologic replicates for both male and female samples were prepared.

### 2.2. Library Construction and High-Throughput Sequencing

Total RNA from each of the six samples was extracted using the SV Total RNA Isolation System (Promega). We assessed the RNA quality using agarose gel electrophoresis and measured RNA integrity using the RNA Nano 6000 Assay Kit of the Agilent Bioanalyzer 2100 system (Agilent Technologies, Santa Clara, CA, USA). Sequencing libraries preparation and high throughput sequencing were generated by Novogene (Beijing, China) following our previous study [27,28,29]. Briefly, mRNA was purified from total RNA with poly-T oligo-attached magnetic beads and fragmented into short pieces. Then, the first-strand cDNA was synthesized using random hexamer primer and second-strand cDNA was then generated. Finally, the paired-end cDNA library was prepared according to the Illumina’s protocols and sequenced on Illumina HiSeq 4000 platform (150 bp paired-end) (Illumina, San Diego, CA, USA). The RNA-seq reads were deposited into the National Center for Biotechnology Information (NCBI) Sequence Read Archive database (Accession No. SRP154651, Supplementary Materials Table S1).

### 2.3. Analysis of RNA-Seq Data

Raw RNA-seq reads were first filtered to delete primer dimers and low-quality bases (Phred quality score lower 20) using the Trim Galore! program (version 0.3.7) (https://www.bioinformatics. babraham.ac.uk/projects/trim_galore/). We only retained paired-end reads from which either end was longer than 50 bp after trimming for subsequent analyses. High quality paired-end reads from each sample were aligned to the transcripts from zebrafish genome annotated by Ensembl (release 97) [30] using Bowtie2 [31];abundances of transcripts(FPKM, Fragments Per Kilobase Million)were estimated using RSEM program (v 1.3.1) [32]. We filtered the genes with FPKM > 1 in at least half of the six samples as transcriptionally active genes for subsequent analyses. The raw read counts for each transcript estimated by RSEM were extracted and then normalized using TMM method to control for differences in sequencing depth among samples, and the differentially expressed transcripts were identified using the edgeR package [33] using a minimal fold change of 2 and an adjusted *p* value cutoff of 0.05. Full lists of differential expression genes can be found in Supplementary Materials Table S2.

### 2.4. Gene Ontology Analysis

Overrepresentation of the gene ontology (GO) terms for upregulated genes between male and female zebrafish olfactory epithelium were identified using Gorilla (http://cbl-gorilla.cs.technion.ac.il/) [34], which allows detection functional overrepresentation in a candidate data set against a list of background genes. We set the false discovery rate (FDR) of 0.001 as our cutoff value and conducted separately for each sex.

### 2.5. Characterization of Alternative Splicing Events (ASEs)

ASEs are divided into five broad categories including skipped exon (SE), alternative 5′ splice site (A5SS), alternative 3′ splice site (A3SS), mutually exclusive exons (MXE) and retained intron (RI) [35]. We used rMATS [36] to detect and count reads that correspond to each of the five types of ASEs. rMATS can identify these ASEs events from a GTF file of annotated transcripts and count the number of reads that correspond to each of the five events described. To identify differential alternative splicing (DAS) events between male and female zebrafish olfactory epithelium, an FDR-adjusted *p* value less than 0.05 was used as threshold for DAS events (Supplementary Materials Table S6).

### 2.6. Data Mining for the Chemosensory Receptor Repertoire

We extracted all the sequences from annotated and automatically predicted paralogs for *or*, *taar*, *ora*/*V1r* and *olfC*/*V2r* genes from the Ensembl zebrafish genome (GRCz11, release 97). We only considered a gene as a putative chemosensory receptor gene for a given family by checking the candidates position within each chemosensory receptor family clade in a phylogenetic analysis. By using this method, we obtained a total of 170 *or*, 126 *taar*, 5*ora*/*V1r* and 57 *olfC*/*V2r* genes.

### 2.7. Phylogenetic Analysis

All of the coding sequences for the chemosensory receptor genes were obtained from Ensembl. The coding sequences for each of the 4 chemosensory receptor gene families were translated into protein sequences, aligned with the program MUSCLE [37] and then back-reversed to their coding sequences alignment. The ML trees were reconstructed by RAxML (version 8.1.17) [38] under the GTRGAMMMAI substitution model with bootstrap support values determined using 1000 replicates.

### 2.8. Quantitative Real-Time PCR (qRT-PCR)

In order to confirm the differentially expressed genes detected by RNA-seq, we further employed quantitative real-time PCR (qRT-PCR) on a subset of genes that among the significantly DEGs between the male and female zebrafish olfactory epithelium. Primers of these genes were designed using the NCBI primer designing tool (http://www.ncbi.nlm.nih.gov/tools/primer). We synthesized the first strand cDNA from 500 ng of total RNA samples using M-MLV Reverse Transcriptase (Promega, Madison, WI, USA) and diluted 1:10 as amplification template. qRT-PCR was performed in a 10-μL volume using the LightCycler^®^ 480 SYBR Green I Master on a LightCycler^®^ 480 II Instrument (Basel, Roche, Switzerland). Thermocycling conditions were 95 °C for 5 min, followed by 45 cycles of 95 °C for 20 s and 58 °C for 25 s, and a melting curve analysis was performed to confirm the primer specificity after amplification. The relative gene expression between male and female zebrafish olfactory epithelium was determined using the comparative CT method [39] and the fold change values were the mean of six biologic replicates from each group.

## 3. Results

### 3.1. Transcriptome Data

A total of six cDNA libraries were constructed in this study. In total, we obtained 23.75, 24.44, 25.65 million reads for male olfactory epithelium and 22.96, 21.70, 22.28 million reads for female olfactory epithelium, respectively. From these, 75.46–77.75% of the reads can be mapped to the annotated regions in zebrafish genome, indicating the good quality of reads (Supplementary Materials Table S1). Overall, we detected a total of 18,658 genes with FPKM > 1 in at least half of the six samples, which were defined as robustly expressed genes. The expression estimates for all annotated transcripts in each replicate were provided in Supplementary Materials Table S2.

**Figure 1:**
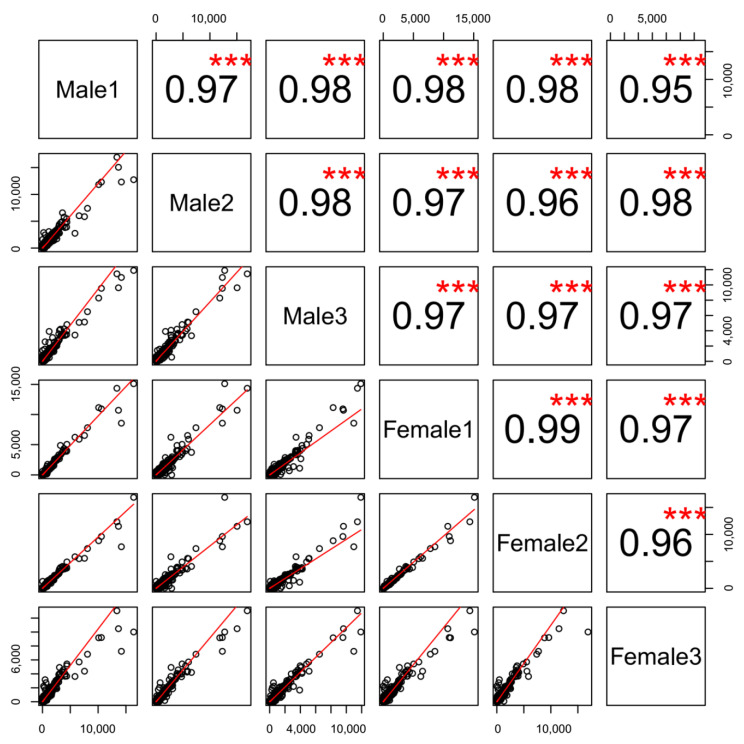
Correlations of gene expression profile between biologic replicates. The three male and three female samples are listed on the diagonal. Above the diagonal the rho value of the Pearson correlation coefficient is indicated. Below are pairwise comparisons between biologic replicates, shown as scatter plots of the abundances of transcripts(FPKM) expression values for all genes. *** means *p-*value > 0.95.

**Figure 2:**
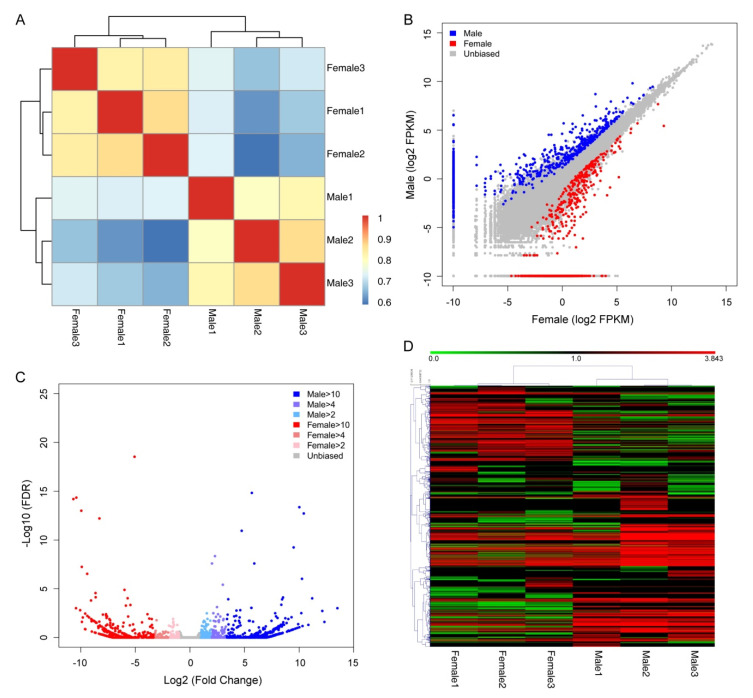
Sexual dimorphism in zebrafish olfactory system. (**A**) Heatmap of cross-correlations of all samples using differentially expressed transcripts; (**B**) pairwise comparison of gene expression abundances between male and female; (**C**) log2-fold change between male and female samples is plotted against their −log10 FDR; (**D**) heatmap of differentially expressed transcripts identified in zebrafish olfactory epithelium.

We first evaluated the variation in gene expression among the three biologic replicate samples for each sex. We found that the correlation values were highly significant between them all (Figure 1, Pearson correlation coefficients of at least 0.95, *p*-value < 2.2 × 10^−16^). Only very small sets of genes are unusually variable among replicates (Figure 1). In general, the OE transcriptomes from the male and the female zebrafish were highly correlated (Figure 2A, Pearson correlation coefficient, *r* = 0.89, *p* < 2.2 × 10^−16^). We therefore averaged the FPKM values for each gene across each sex. In the olfactory epithelium from both male and female, a few gene are extremely highly expressed. For example, in male 312 most abundant genes account for almost 50% of the fragments and in female 333 most abundant genes account for almost 50% of the fragments obtained from the tissue (Supplementary Materials Table S3). Moreover, a total of 295 genes were found to be shared between male and female most abundant genes. These results were generally consistent with the patterns found in mouse olfactory transcriptomes [40].

### 3.2. Sex Dimorphism in Zebrafish Olfactory Epithelium Expression Profiles

To assess whether transcriptional differences in the zebrafish olfactory epithelium can account for sex-specific responses to olfactory cues, we examined their sexually dimorphic gene expression profiles. We found that the overall transcriptomes are highly similar between male and female zebrafish olfactory epithelia (Figure 2A,B). Briefly, we detected a total of 713 transcripts showing higher expression level in male olfactory epithelium and 605 transcripts showing higher expression level in female olfactory epithelium, respectively (Figure 2C). However, only 68 and 53 transcripts remained significantly differential expression between male and female olfactory epithelium after applying a false discovery rate (FDR) threshold of 0.05 (Figure 2D and Supplementary Materials Table S4). Among these, some genes identified to be differentially expressed between sexes are expected to be involved in response to olfactory cues (Table 1), such as gene *OR132-2* (odorant receptor, family H, subfamily 132, member 2), *OTX1* (orthodenticle homeobox 1) and *OR115-10* (odorant receptor, family F, subfamily 115, member 10). However, whether these differentially expressed olfactory receptor genes can detect sex relative pheromones needs to be verified in further functional experiments.

**Figure 3:**
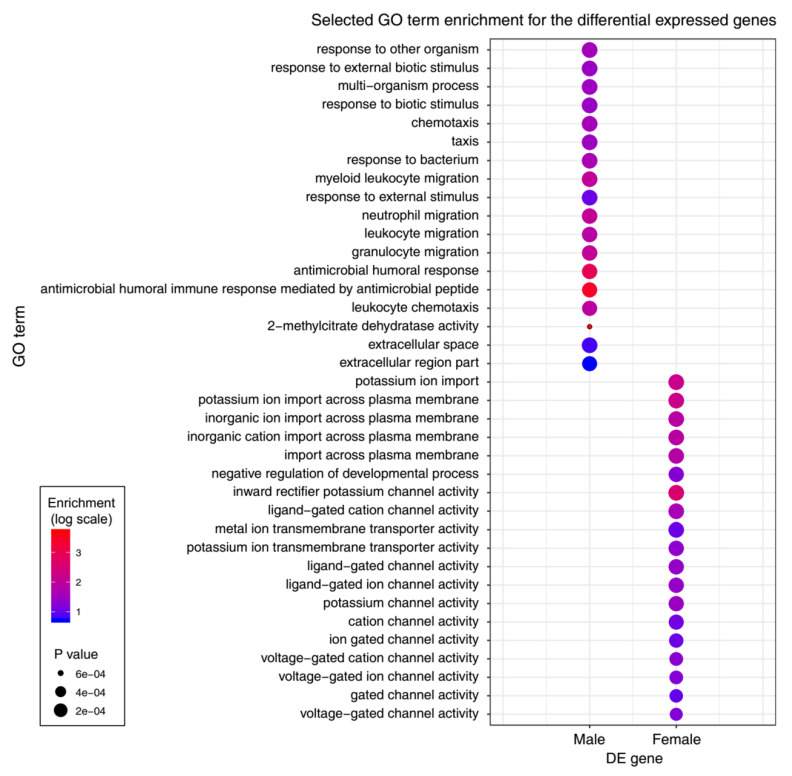
Gene ontology (GO) terms associated with differentially expressed genes between male and female zebrafish olfactory epithelium.

To gain further insights into the biologic processes that differed between male and female zebrafish olfactory epithelium, we performed a GO enrichment analysis with the upregulated genes detected in male and female zebrafish olfactory epithelium (Supplementary Materials Table S5). We found that gene ontology biologic process terms associated with response to external stimulus were highly overrepresented among the differentially expressed genes (DEGs) with male-biased expression (Figure 3 and Supplementary Materials Table S5), including response to other organism (GO:0051707), chemotaxis (GO:0006935) and taxis (GO:0042330). However, a significantly different pattern was found in the enriched GO terms for female olfactory epithelium upregulated genes. For example, female olfactory epithelium upregulated genes were enriched in ion import, such as potassium ion import (GO:0010107), inward rectifier potassium channel activity (GO:0005242) and ligand-gated channel activity (GO:0022834). Taken together, these results indicated that there are significant differences in the upregulated genes between male and female zebrafish olfactory epithelium.

### 3.3. Alternative Splicing between the Two Sexes of Zebrafish Olfactory Epithelium

**Figure 4:**
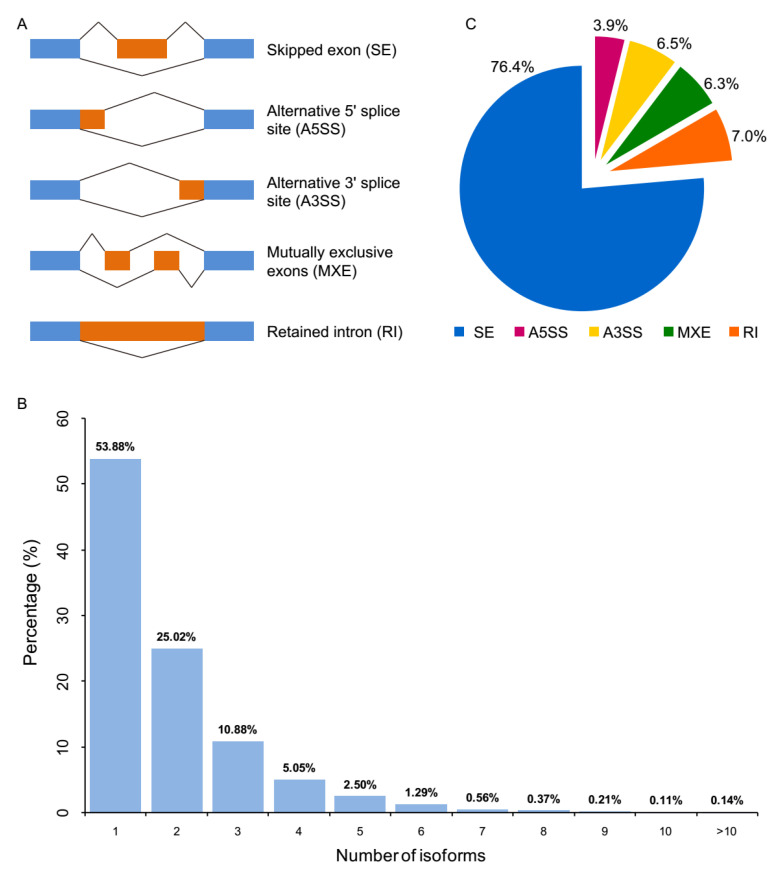
Alternative splicing events (ASEs) between male and female zebrafish olfactory epithelium. (**A**) Schematic representation of five basic types of alternative splicing events. Alternative exons are shown as orange boxes and flanking constitutive exons are shown as blue boxes; (**B**) distribution of isoform numbers for genes in zebrafish genome; (**C**) pie chart showing the percentage distribution of ASEs.

To examine the influence of sex on alternative splicing regulation in zebrafish olfactory epithelium, alternative splicing events (ASEs) including skipped exon (SE), alternative 5′ splice site (A5SS), alternative 3′ splice site (A3SS), mutually exclusive exons (MXE) and retained intron (RI) (Figure 4A) between male and female zebrafish olfactory epithelium were identified using rMATS [36].

Roughly 46% of genes have 2 detectable isoforms in zebrafish genome (Figure 4B). Among them, we detected a total of 15,193 ASEs, which were distributed in 7585 genes. The most abundant ASEs were skipped exon, accounting for 76.4% of all ASEs, followed by retained intron (7.0%), alternative 3′ splice site (6.5%), mutually exclusive exons (6.3%) and alternative 5′ splice site (3.9%) (Figure 4C). Using *p* value cutoff of 0.05, differential ASEs were identified between the two sexes of zebrafish (Supplementary Materials Table S6). A total of 86 significantly differential ASEs were identified between the male and female zebrafish olfactory epithelium, which included 50 SE, 4 A5SS, 17 A3SS, 6 MXE, and, 9 RI (Table 2). For example, *maptb*, which is expressed in the developing central nervous system [41], was found to generate different isoforms by sex. *stxbp5l*, which is involved in syntaxin binding, also showed differential isoforms between sexes, which was identified as sexually dimorphic gene between cattle and rat species [42].

### 3.4. The Chemosensory Receptor Repertoires

As in mouse olfactory sensory neurons (OSNs), each of the OSNs in mature zebrafish randomly expressed only one olfactory receptor [43,44,45]. Thus, the expression level of any given olfactory receptor within the olfactory epithelium will be low. Consistent with this prediction, among the 170 *or*, 126 *taar*, 5*ora*/*V1r* and 57 *olfC*/*V2r* genes in the zebrafish genome, we detected 147 out of 170 *or*, 104 out of 126 *taar*, 4 out of 5*ora*/*V1r* and 57 out of 57 *olfC*/*V2r* genes expressed in either male or female olfactory epithelium with FPKM > 1, confirming that the chemosensory receptors are mainly expressed in the olfactory epithelium (Supplementary Materials Table S7). However, almost all (90%) of these chemosensory receptor genes were found to be very lowly expressed in the zebrafish olfactory epithelium transcriptomes (FPKM < 15), which is consistent with the results from mice [46].

**Figure 5:**
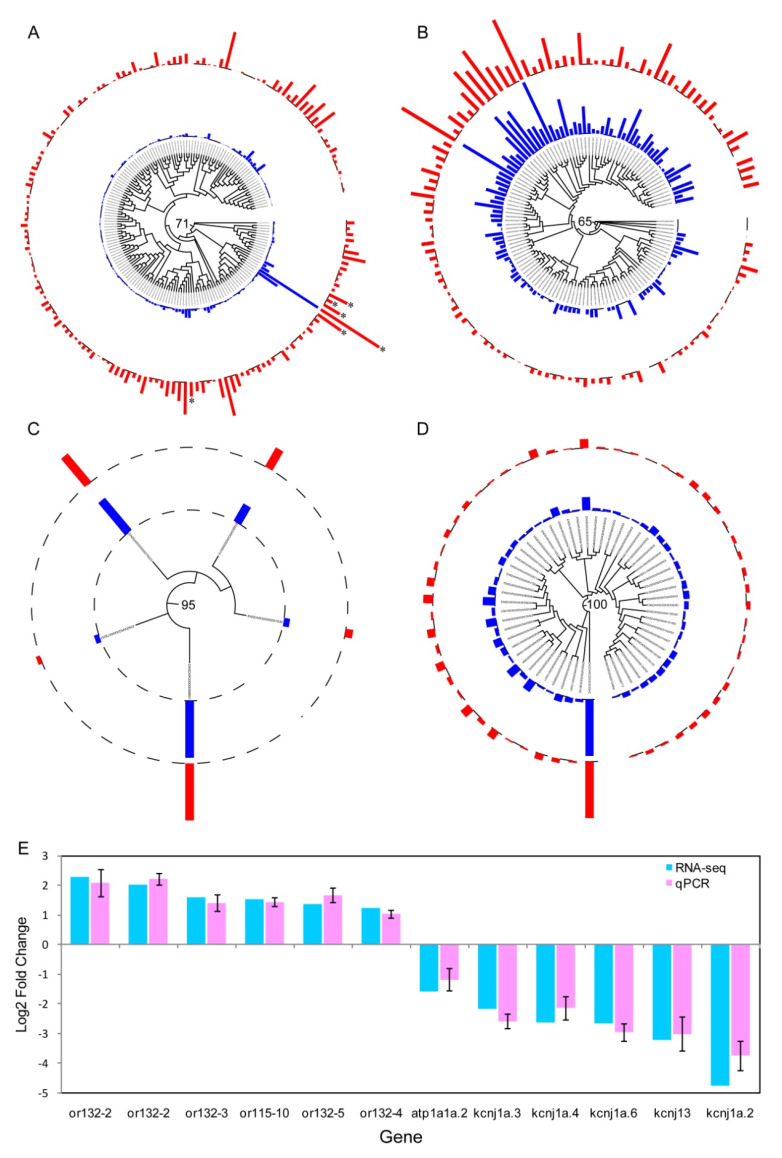
Expression pattern of the chemosensory receptor genes in the zebrafish olfactory epithelium. Mean FPKM expression values across the three samples between male (blue) and female (red) for each of the *or* (**A**), *taar* (**B**), *ora*/*V1r* (**C**) and *olfC*/*V2r* (**D**) genes. Phylogenetic trees were reconstructed using RAxML (version 8.1.17) under the GTRGAMMMAI model with bootstrap support values determined using 1000 replicates. * means differentially expressed genes. Bootstrap values for basal nodes are provided; (**E**) validation of RNA-seq data using quantitative real-time PCR (qRT-PCR).

To further assess whether there are differences in chemosensory receptor gene expression levels between the two sexes in zebrafish, we compared the expression levels of chemosensory receptor genes between male and female zebrafish olfactory epithelium transcriptome. From this analysis, we made the following interesting observations. First, the relative receptor abundance level vary greatly between each member, suggesting that each member of the chemosensory receptor genes was expressed asymmetrically. Some members have high expression levels, whereas other members expressed with very low levels (Figure 5). Second, almost all of the chemosensory receptor genes expressed with no differences between male and female zebrafish olfactory epithelium, with only six *or* genes showing differentially expressed between the two sexes (Supplementary Materials Table S7). Third, all of the 6 *or* genes (Table 1) that showed sexual preference were expressed at a higher level in the male olfactory epithelium than in females, which is generally consistent with the results from mice [40,46]. In order to verify the expression results from RNA-seq, we further employed qRT-PCRto measure relative mRNA levels for a total of 12 candidate genes. Our results demonstrated that the expression patterns for these genes were highly consistent between RNA-seq and qRT-PCR (Figure 5E), suggesting the reliability of our RNA-seq datasets.

## 4. Discussion

During the last few decades, chemosensory receptor gene families have received extensive attention across various organisms, including humans [45,47,48,49], mice [40,46,50], fish [28,51,52,53] and insects [21,54,55,56,57]. However, it remains still unclear whether male and female individual possesses the same ability for odorant-sensing. In the present study, we reported the transcriptional profiles of the olfactory epithelium from the male and female zebrafish obtained by RNA-seq. By comparing the expression levels of genes from three different biologic replicate samples for each sex, we obtained highly correlated samples, which suggested that the subsequent differential expression analyses were reliable.

Sexual dimorphisms in behaviors have been widely observed in the olfactory systems in both mice [50,58] and flies [59]. Although previous studies have suggested that sexually dimorphic behaviors between sexes could be influenced by sensory input such as olfactory cues [60], it is unclear whether differences in gene expression in olfactory epithelia can underlie sexually dimorphic behaviors in fish [40]. Our results showed that the overall transcriptional profiles of zebrafish olfactory epithelium between sexes are highly similar. However, we indeed detected a few genes differentially expressed between male and female zebrafish, whose roles may be associated with odorant-sensing. Moreover, the functional enrichment analyses of the differentially expressed genes were also involved in response to external biotic stimulus. Therefore, all these results from our study suggested that differences in gene expression in the olfactory epithelium between sexes in zebrafish may play a role in odorant sensing to some extent.

However, when we focused on the chemosensory receptor genes that are directly binding to the odorants in surrounding environment, our results suggest that the olfactory receptor genes may not all be involved in behavioral differences between males and females. Although almost all of the chemosensory receptor genes can be detected to be expressed in the olfactory epithelium of zebrafish, most them displayed similar expression levels between sexes, with only six *or* genes showing differentially expressed between the two sexes. Considering the existence of nearly 358 chemosensory receptor genes in zebrafish genome, the six differentially expressed *or* genes should play minor roles in the dimorphic behaviors between male and female zebrafish [61,62]. Therefore, the sexually dimorphic behavioral responses to odorants in zebrafish are unlikely to be solely accounted for by transcriptional differences at the level of detection [40].

Interestingly, several functional studies have identified several olfactory receptor ligands in fish [10,11,12,13,14,15,16]. For example, Yabukiet al. reported that *or114-1* and *or114-2* in the group β *or* genes are the key olfactory receptors for the sex pheromone prostaglandin F_2α_ to mediate male courtship behavior in zebrafish [16]. However, none of these olfactory receptor genes were found to be significantly differentially expressed in the olfactory epithelium between male and female in our RNA-seq results. It should be noted that differential expression of peripheral odorant receptors is not the only way to generate sex-specific behavior, as higher neuronal circuits could be different between males and females. Therefore, whether the six differentially expressed *or* genes can play crucial roles in sexually dimorphic behaviors is still uncertain and need to be confirmed by further functional studies in future.

## 5. Conclusions

In this study, we performed a transcriptomic analysis on the olfactory epithelia of both male and female zebrafish using high-throughput RNA sequencing. We found the olfactory transcriptomes between males and females are highly similar, with only few genes displaying differentially expressed. Most of chemosensory receptor genes showed evidence of expression in the zebrafish olfactory epithelia, with a higher level of expression in males than in females. Collectively, these results provide a comprehensive catalog of the genes mediating olfactory perception and pheromone-evoked behavior in fish.

## Figures and Tables

**Table 1:** List of selected differentially expressed genes.

Gene ID	Gene Name	Gene Description	Log2(Male/Female)	*p-*Value	FDR
ENSDARG00000105762	or132-2	odorant receptor, family H, subfamily 132, member 2	2.295	1.07 × 10^−12^	4.48 × 10^−9^
ENSDARG00000094515	or132-2	odorant receptor, family H, subfamily 132, member 2	2.021	7.02 × 10^−12^	2.56 × 10^−8^
ENSDARG00000035048	or115-10	odorant receptor, family F, subfamily 115, member 10	1.546	3.02 × 10^−6^	0.003
ENSDARG00000105835	or132-3	odorant receptor, family H, subfamily 132, member 3	1.606	1.81 × 10^−5^	0.012
ENSDARG00000056277	or132-5	odorant receptor, family H, subfamily 132, member 5	1.373	4.86 × 10^−5^	0.024
ENSDARG00000105719	or132-4	odorant receptor, family H, subfamily 132, member 4	1.232	5.84 × 10^−5^	0.027
ENSDARG00000094992	otx1	orthodenticle homeobox 1	10.205	2.68 × 10^−6^	0.003
ENSDARG00000062593	stox1	storkhead box 1	−10.404	2.91 × 10^−19^	4.47 × 10^−15^
ENSDARG00000075271	rapgef5a	Rap guanine nucleotide exchange factor (GEF) 5a	−8.647	1.13 × 10^−8^	2.75 × 10^−5^
ENSDARG00000060610	pcdh7b	protocadherin 7b	−8.962	2.51 × 10^−5^	0.016

**Table 2:** Of selected differential alternative splicing events.

Gene ID	Gene Name	Gene Description	ASEs Type	*p-*Value	FDR
ENSDARG00000087616	*maptb*	microtubule-associated protein tau b	SE	1.79 × 10^−8^	6.91 × 10^−5^
ENSDARG00000006383	*stxbp5l*	syntaxin binding protein 5-like	SE	2.26 × 10^−6^	0.002
ENSDARG00000019208	*camsap1a*	calmodulin regulated spectrin-associated protein 1a	SE	0.0001	0.04
ENSDARG00000041736	*hsdl1*	hydroxysteroid dehydrogenase like 1	SE	0.0001	0.03
ENSDARG00000055825	*celsr3*	cadherin, EGF LAG seven-pass G-type receptor 3	A5SS	6.9 × 10^−6^	0.004
ENSDARG00000014577	*rhpn2*	rhophilin, Rho GTPase binding protein 2	A5SS	3.55 × 10^−5^	0.01
ENSDARG00000074255	*micu3b*	mitochondrial calcium uptake family, member 3b	MXE	1.44 × 10^−7^	6.92 × 10^−5^
ENSDARG00000077818	*nrg2a*	neuregulin 2a	MXE	0.0002	0.03
ENSDARG00000086103	*slc37a1*	solute carrier family 37 (glucose-6-phosphate transporter), member 1	MXE	0	0
ENSDARG00000025338	*hagh*	hydroxyacylglutathione hydrolase	RI	5.9 × 10^−7^	0.0003

## Supplementary Materials

The following are available online at https://www.mdpi.com/2073-4425/11/6/592/s1, Table S1: Summary of the RNA-seq datasets in olfactory mucosae from male and female zebrafish, Table S2: Expression estimates in the zebrafish olfactory epithelium. A dataset containing the raw and normalized expression values for all the genes in the zebrafish olfactory epithelium, Table S3: 312 and 333 most abundant genes account for 50% of the fragments obtained from the tissue for male and female, Table S4: Differential expression analysis between male and female samples. Genes with an FDR < 0.05 were considered significant, Table S5: Functional enrichment analysis for differentially expressed genes, Table S6: Differential alternative splicing events between male and female zebrafish olfactory epithelium, Table S7 Expression level of the chemosensory receptor genes.
